# Effectiveness of Complementary and Integrative Approaches in Promoting Engagement and Overall Wellness Toward Suicide Prevention in Veterans

**DOI:** 10.1089/acm.2020.0245

**Published:** 2021-03-31

**Authors:** Amanda Vitale, Lauren Byma, Shengnan Sun, Evan Podolak, Zhaoyu Wang, Sharon Alter, Hanga Galfalvy, Joseph Geraci, Erik Langhoff, Heidi Klingbeil, Rachel Yehuda, Fatemeh Haghighi, Ann Feder

**Affiliations:** ^1^ James J. Peters VA Medical Center, Bronx, NY, USA.; ^2^ Icahn School of Medicine at Mount Sinai, New York, NY, USA.; ^3^ Department of Psychiatry, Columbia University, New York, NY, USA.

**Keywords:** suicide prevention, depression, meditation, mind–body, nutrition, sleep, complementary and integrative health

## Abstract

***Objective:*** Suicide is a major public health problem, specifically among U.S. veterans, who do not consistently engage in mental health services, often citing stigma as a barrier. Complementary and Integrative Health (CIH) interventions are promising alternatives in promoting patient engagement and further, they may play a critical role in transitioning people into mental health care. Toward this goal, the Resilience and Wellness Center (RWC) was developed to break through the stigma barrier by addressing risk factors of suicide through multimodal CIH interventions via cohort design, promoting social connectedness and accountability among participants.

***Design:*** This is a program evaluation study at a large urban VA medical center, where assessments were evaluated from pre- to post-program completion to determine the effectiveness of an intensive multimodal CIH 4-week group outpatient intervention for suicide prevention.

***Outcome measures:*** Primary outcomes measured included group connectedness, severity of depression and hopelessness symptoms, suicidal ideation, sleep quality, and diet. Secondary outcomes included measures of post-traumatic stress disorder (PTSD), generalized anxiety severity stress/coping skills, pain, and fatigue.

***Results:*** The RWC showed high participant engagement, with an 84%–95% attendance engagement rate depending on suicide risk history. Data from 15 cohorts (*N* = 126) demonstrate favorable outcomes associated with participation in this comprehensive program, as evidenced by a reduction in suicidal ideation, depression, and hopelessness, but not sleep quality and diet. In addition, in a subset of veterans with a history of suicidal ideation or attempt, significant improvements were noted in pain, PTSD/anxiety symptoms, and stress coping measures.

***Conclusions:*** The RWC shows that an intensive complement of CIH interventions is associated with a significant improvement with high veteran engagement. Findings from this program evaluation study can be used to aid health care systems and their providers in determining whether or not to utilize such multimodal CIH integrated interventions as an effective treatment for at-risk populations as a part of suicide prevention efforts.

## Introduction

A critical problem in suicide prevention is a lack of engagement in mental health services, with stigma being an oft-cited barrier,^1^ especially among veterans, a vulnerable population at high risk for suicide. Despite making up only 7.9% of the population, veterans account for 13.5% of all deaths by suicide.^2^ Veterans typically have multiple comorbid psychiatric diagnoses, with complex trauma histories (e.g., deployment-related trauma,^3–6^ ongoing current life stressors,^7–11^ and antecedent childhood adversity^9,12^), which contribute to this heightened suicide risk. Evidence-based practices for the treatment of patients at risk of suicide and associated comorbidities, such as cognitive behavior therapy (CBT), acceptance and commitment therapy, and dialectical behavioral therapy, have been shown to be effective in research settings.^13–18^ There is, however, a growing recognition in suicide prevention of the evidence-practice gap when translating suicide prevention psychotherapy intervention research into real-world clinical practice, with only limited effectiveness.^19^ To address this gap, approaches, including diversified delivery models^19^ and increased risk assessment screening and training,^20^ have been proposed; however, the problem remains that not all who are in need of mental health services are willing to engage in these services. For instance, therapeutic approaches to address suicidal behavior and comorbid psychiatric disorders, particularly among veterans, show limited patient engagement due to patients' aversion to reliving painful or upsetting memories (such as cognitive processing therapy and prolonged exposure therapy used for PTSD^21^), or associated stigma with treatment.^22–24^

There is a critical need to develop and evaluate alternative treatment modalities for effectively engaging at-risk veterans. The recent publication of the National Strategy for Preventing Veteran Suicide^25^ has recognized the importance of building resilience and promoting overall wellness toward suicide prevention. In line with this national priority, Complementary and Integrative Health (CIH) approaches provide the opportunity to expand the repertoire of therapeutic interventions for these at-risk populations. Veterans have shown willingness and interest to engage in CIH therapies,^26^ with data from a 2017 national sample showing that more than 52% of veterans have used some form of complementary services, and 84% are interested in trying or learning more about CIH approaches.^27^

Although the impact of specific CIH interventions (e.g., mindfulness, acupuncture) has been investigated separately with significant effects,^28,29^ to our knowledge none has assessed a combined multimodal CIH approach in determining the effectiveness of CIH treatments toward the goal of mitigating suicide risk. The Resilience and Wellness Center (RWC) at an urban VA Medical Center was created to provide multimodal CIH therapeutic interventions in a group setting for at-risk veterans currently receiving care at the VA, utilizing a variety of CIH life skills interventions, such as: physical activity, diet/nutrition, creative expression, acupuncture, sleep hygiene, and stress management. This CIH approach aims at reducing symptoms associated with comorbid psychiatric disorders, and confluence of suicide risk factors including poor sleep quality,^30–33^ diet,^34–36^ stress,^7,9^ chronic pain,^37^ and social isolation.^10,38,39^ We present here the results of a program evaluation examining the effectiveness of the RWC to determine whether these multimodal CIH interventions cumulatively exert significant benefits on this vulnerable population, through participant engagement and measure of effectiveness and through assessments of mental and general health outcomes: depression, anxiety, stress and coping skills, pain, sleep quality, and diet, as well as suicidal ideation. Assessments pre- and post-RWC program completion were investigated. The goal was to assess the utilization of this transdiagnostic approach (i.e., inclusive of multiple comorbid psychiatric and medical conditions) to improve health and well-being in the setting of usual medical care, allowing for evaluation of the “real-world” benefits of this multimodal CIH approach in reducing suicidal ideation and associated symptoms.

## Materials and Methods

The RWC program evaluation was reviewed and determined by the Institutional Review Board and the Quality Improvement Executive Committee of the VA hospital to be exempt from IRB review, and it was approved by the Quality Improvement Executive Committee; as such, we utilized deindentified data from participant responses and did not perform informed consent, provide participants with monetary compensation, or register this program evaluation as a clinical trial.

### Design

Launched in October 2018, the RWC was developed by a team of clinicians and researchers, with the aim of providing CIH interventions that target key risk factors of suicidal behavior (see Table 1 for detailed program curriculum and frequency). To operationalize RWC goals and gain support from leadership and program staff, the curriculum, activities, participation model, and outcome metrics were developed in collaboration with these staff. The VA staff with CIH skills and training were approached to lead one to three classes throughout the week, with workload going to their service line, which includes Rehabilitation Medicine, Mental Health, Nutrition, Chaplaincy, and Research. The transcendental meditation (TM) class was offered by a TM Instructor from the David Lynch Foundation, which had already established TM instruction opportunities for veterans at the medical center before RWC inception. Although individual stand-alone CIH treatments are routinely offered through these VA services, the RWC was developed as an intensive month-long multimodal CIH intervention intended to be immersive and social (via cohort design). Inclusion is transdiagnostic, targeting at-risk vulnerable veterans who are isolated and experiencing ongoing environmental stressors, and who would like to learn new coping strategies and attempt to make significant lifestyle changes. Participants were clinician-referred and were subsequently contacted by RWC staff to confirm understanding and willingness to attend a 5 day per week, 3-h daily, 4-week program AND to be mindful to avoid scheduling conflicts while continuing their other treatments as usual. The RWC program engagement and outcome has been evaluated for 15 independent cohorts to date (*N* = 126), with data collected at program inception for primary outcome measures, and a subset of these participants (*N* = 82) across 9 cohorts providing secondary outcome measures (additional to the primary outcomes).

**Table 1.** Description of Complementary and Integrative Health Interventions and Number and Amount of time the Interventions are Delivered

Interventions	No. of times intervention offered	Total no. of minutes the intervention offered
Mind–Body		
EFT: Participants learn how to tap on specific acupressure points to relieve stress and uncomfortable physical sensations while identifying problems and core issues.	4	240
Transcendental Meditation: is a consistent practice, found to be effective for reducing stress and stress-related disorders. Participants are taught to enter a state of relaxed awareness of thoughts and physical sensations by continuously thinking the instructor-chosen mantra. Veterans are encouraged to practice outside the course to extend benefits.	5	300
Mindful Awareness: teaches veterans to be aware of their physical and emotional responses and environment in the present moment without judgment. The veterans engage in breath, body, movement, and sound awareness experiences for a duration of 5 min before the start of each day's classes, to help veterans transition into and bring their focus to the daily classes.	16	80
Expressive Arts		
Narrative Therapy: guided by a clinician, members freely write on a given topic or a response to a poem/text, and then share what they have written with the group with the goal of cultivating a larger understanding of group commonalities and generating solutions to problems raised. Narrative Therapy targets at-risk veterans by providing a venue for self expression, interpersonal understanding, sharing, and personal growth.	4	240
Music Therapy: The goal is to increase personal insight into the veteran's inter- and intrapersonal communication and health behaviors with the intention of learning strategies for using music independently to manage anxiety, depression, anger, and chronic pain. It emphasizes a dynamic combination of passive and active interventions, including guided imagery, songwriting, singing/playing familiar music, lyric discussion, and thematic percussion improvisation.	4	240
Exercise and Physical Wellness		
Aerobic Line Dancing: movement-focused dance in which a group of veterans execute choreographed steps at the same time, as demonstrated by the physical therapist instructor. This unique form of exercise combines physical benefits with social engagement, encouraging veterans to not only keep fit but also learn to master a dance and build relationships.	12	720
Many of the participating veterans expressed familiarity with soul line dancing from community and/or family events.		
Yoga: aims at increasing bodily awareness, relieving stress, reducing muscle tension, strain, and inflammation, sharpening attention, and calming the central nervous system. Tailored to the physical capabilities of veteran participants, chair yoga allows participants to get the full mental and physical benefits of yoga with a reduced chance of strain or physical risk.	4	240
Acupuncture: The only one-on-one session provided to the participants. Veterans are evaluated by a certified acupuncturist, who then offers individualized acupuncture treatment for the duration of the program; although its primary focus is on pain, it also targets secondary symptoms of depression, anxiety, poor sleep, and associated quality of life issues.	4	120
Sleep Hygiene		
Sleep Hygiene: teaches the development of healthy sleep habits/spaces at home. Veterans learn about what factors foster and maintain high-quality sleep and about habits that might sabotage the sleep process. It also introduces veterans to options for more focused behavioral treatment interventions (e.g., CBT for insomnia).	4	240
Spirit and Soul		
Spirituality: Based on Viktor Frankl's Meaning Centered Logotherapy and Existential Analysis,^1^ this class provides practical techniques for addressing crises by fostering personal meaning and purpose using an integrated Mind-Body-Spirit Approach that empowers veteran participants.^2^	4	240
Nutrition		
Nutrition and Cooking: teaches the important role that healthy cooking skills play in promoting resilience and wellness through evidence-based approaches to decrease chronic pain and inflammation and improve mood and energy. Veteran participants try new recipes, improve their basic cooking skills, learn about dietary modifications, and experiment with a variety of ingredients. This multifaceted course is an interactive, dynamic, and personalized cooking experience. Veterans are taught sustainable ways to incorporate these recipes and cooking techniques into their daily meal planning to support healthy lifestyle behaviors.	8	240
Life Skills		
Financial Literacy: included in this is an area of significant stress that focuses on topics such as managing personal budgets and investing, focusing on practical budgeting as a means to independence.	4	240
Interpersonal Effectiveness: teaches assertiveness techniques so participants can ask for their wants while balancing the need for healthy relationships and self-respect. It is grounded in DBT-based protocols and emphasizes the use of coping skills and the setting of healthy boundaries.	4	240

CBT, cognitive behavior therapy; DBT, dialectical behavioral therapy; EFT, Emotional Freedom Technique.

### Participants

Data are presented on 15 cohorts, with assessments performed on 126 participants consisting of 87 males and 39 females, ranging in age from 28 to 77 years. The racial makeup of the participants consisted of 13% identifying as white non-Hispanic, 25% white Hispanic, 47% black non-Hispanic, 13% black Hispanic, and 2% Asian/Pacific Islander.

### Multimodal CIH treatment intervention

Interventions were employed based on existing in-house expertise and availability of staff and practitioners from various service lines in the medical center, as well as the external TM practitioner, targeting specific CIH domains and risk factors associated with suicidal behavior (see description CIH modalities in Table 1, and representative week's schedule of RWC programing in Supplementary Table S1).

#### Social isolation

The RWC cohort design aims at attenuating social isolation, including active participation and conversation with instructors and each other to foster cohesion and build a sense of community among participants. As Joiner posited in the Interpersonal Theory of Suicide, thwarted belongingness (the lack of belonging to a stable social structure of family, friends, or other valued group) is a large part of the drive to attempt suicide,^40^ and indeed, suicide in veterans is more common in those who are divorced, widowed, or single.^41^ Throughout the RWC group programing, developing a sense of community not only combats social isolation but also presents a supportive environment to address military-related traumas. Building social connections through shared experiences promotes group cohesion toward the goal of reducing feelings related to isolation and loss of social connectedness.^42^

#### Mind–body

Mind–body interventions include Emotional Freedom Technique (EFT), TM, and brief mindful awareness exercises. The EFT uses acupressure points and cognitive behavioral therapy techniques for daily stress reduction,^43,44^ and TM, a form of silent mantra meditation wherein the teacher gives the student a Sanskrit mantra to think continuously during meditation, is a widely used strategy effective for reducing daily stress and stress-related disorders comorbid with suicidal behaviors and predictors in veterans, including elevated cortisol levels,^45^ hypertension,^46^ PTSD,^47–49^ depression,^45,50,51^ and insomnia.^52^

#### Expressive arts

The Expressive Arts therapeutic framework allows individuals to express underlying psychological turmoil, and it has used in populations with psychiatric disorders, trauma, and those who engage in nonsuicidal self-injury.^53–55^ Narrative Therapy and Music Therapy interventions offered in RWC program have been previously shown to reduce depressive and PTSD symptoms comorbid with suicidal behavior^56–64^ and foster self-expression to gain insight and promote inter- and intrapersonal communication.^65^

#### Exercise and physical wellness

Exercise and Physical Wellness interventions include aerobic line dancing and yoga (which has been demonstrated to increase bodily awareness, relieve stress, reduce muscle tension, strain, and inflammation, sharpen attention and concentration, and calm the central nervous system^66–69^) to promote physical wellness among participants. Growing evidence confers an association between physical activity and reduction in suicidal ideation.^70–72^ In addition, acupuncture therapy is offered to target depression, anxiety, poor sleep, acute, and chronic pain symptoms associated with suicide risk.^73–79^

#### Sleep hygiene

The Sleep Hygiene intervention teaches participants about what factors foster and maintain healthy sleep habits. Sleep disturbances are recognized as an upstream risk factor for suicide ideation and behavior independent of comorbid psychiatric disorders.^80–83^ Indeed, sleep disturbances, particularly insomnia and nightmares, are identified in the top 10 indicators of suicide warning signs by the Substance Abuse and Mental Health Services Administration.^84^

#### Diet and nutrition

The Diet and Nutrition intervention includes cooking demonstrations with taste-testing, and presentations on a healthy diet. A growing body of research has identified the importance of diet in suicide risk, with a study of 7631 reporting that female attempters ate fewer daily servings of fruit and male attempters tended to eat fewer daily servings of vegetables, suggesting a link between attempt status and poor diet.^85^ Further, a low dietary intake of polyunsaturated fatty acids (PUFAs), specifically omega-3 PUFAs, confers increased suicide risk, with lower levels of PUFAs in blood plasma being associated with self-harm behavior,^86^ and predictive of future suicide attempts.^87^

### Measures

As part of outcome monitoring for patient-centered and measurement-based care,^88,89^ we used validated self-report instruments to assess mental and general health pre- and post-RWC program completion, including depressive symptoms, hopelessness, anxiety, and perceived stress levels—associated risk factors for suicidal behavior—as well as physiological measures such as pain, sleep quality, daytime sleepiness, and diet.

#### Primary outcome measures

(1) Depression/Hopelessness: Included PHQ-9 (Patient Health Questionnaire-9^90^), BDI (Beck Depression Inventory^91^), and BHS (Beck Hopelessness Scale^92^), with high scores reflecting greater depressive and hopelessness symptoms. The BDI is a 21-question measure assessing depression severity, a key risk factor in future suicidal behavior,^93^ and demonstrates high internal consistency and content validity.^94^ In tandem with the BDI, the PHQ-9 is a 9-question clinical tool for evaluation of depressive and suicidal symptoms.^95^ Both questionnaires show similar responses to change during the course of treatment for depression symptoms.^96^ The BHS is a 20-question measure of the construct of relative hopelessness and anticipation about the future,^97^ since hopelessness is associated with suicide risk.^98^
(2) Suicidal Ideation: Because the RWC is not a psychotherapeutic intervention, suicide-specific instruments were not administered to avoid triggering suicidal thoughts. However, improvement in suicidal ideation was assessed through the ideation items in both PHQ-9/BDI. These responses were reviewed, and participants reporting to have suicidal ideations were discreetly approached and assessed for safety to determine whether they had any plan and/or intent for self-harm, and none were determined to be in need of a higher level of care to manage risk.
(3) Sleep: Included PSQI (Pittsburgh Sleep Quality Index), a self-report assessment of seven domains of sleep,^99^ with high scores corresponding to poor sleep quality.
(4) Diet: Included HDQ (Healthy Diet Questionnaire), a 12-question measure assessing frequency of and diversity of dietary choices, including fruits, vegetables, and sweets,^100^ with lower scores representing poorer diet.
(5) Group Cohesion Scale: The Group Cohesion Scale-Revised is a 25-question measure assessing group connectedness and dynamics, including group bonding, avoidance of conflict, and preference for groups.^101^ This scale has been used to assess the interconnectedness of participants within group psychotherapy.^102^
(6) Participant Engagement: Included recording of daily attendance throughout the 4-week program, culminating with a graduation ceremony celebration and certificate of completion, including family and friends.

#### Secondary outcome measures

(1) Anxiety and stress: Included PCL (PTSD Checklist^103^), BAI (Beck Anxiety Inventory^104^), PSS (Perceived Stress Scale^105^), and MoCS Scale (Measure of Current Status Scale^106^), with higher scores representing greater anxiety/stress symptoms, except for the MoCS Scale where higher values correspond to greater stress coping skills. The PCL is a 21-question measure that assesses PTSD symptom clusters, including avoidance behaviors, hypervigilance, and re-living of traumatic events.^103^ The BAI is a 21-question measure of physical and psychological symptoms of anxiety. The PSS is a 10-item measure covering the stress experienced from current life events, which has been validated in populations with suicidal thoughts and behaviors.^107^ The MoCS Scale is a 13-item questionnaire assessing the degree to which an individual utilizes coping behaviors, which has been used to gauge mindfulness and stress coping skills during interventions.^40^
(2) Pain and Daytime sleepiness: Include DVPRS (Defense Veterans Pain Rating Scale^108^). This is a 4-item military-specific measure of pain's interference in daily life. The ESS (Epworth Sleepiness Scale^109,110^) is a 7-item measure of sleepiness and fatigue during waking hours. Higher scores reflect greater pain/fatigue.

For measures of patient demographic factors, including age, sex, race, ethnicity, no-show rates, and history of suicidal ideation/attempt, information was obtained from veterans' electronic medical records (EMRs). The information on suicide ideation/attempt history was used to measure the effectiveness of RWC across the spectrum of suicide risk severity. Also, at RWC completion, participants completed program experience evaluations, providing participant feedback.

### Data analysis

Analyses were performed by using R 3.6.1. Three group comparisons were performed by using Kruskal-Wallis rank-sum test for continuous variables, and Chi-Square test or Fisher's exact test for all the count variables. *Post hoc* pair-wise Wilcoxon rank-sum test or Fisher's exact test with Bonferroni correction was applied when there was a significant three-group comparison result. For quantitative assessment scales and subscales, the differences between pre- and post-assessment totals/subscales were calculated (delta = post − pre), with outliers winsorized and censored to the nearest nonoutlier values within each diagnostic group. One sample *t* test was used when analyses met normality assumptions, and when violated, the Wilcox signed-rank test was used. Benjamini-Hochberg correction was applied to adjust for multiple comparisons. Cohen's *d*^111^ was used to measure RWC treatment effects, when normality assumptions were met. To exam the weekly attendance number over time, the cumulative link mixed model with subject as the random intercept was fitted.

## Results

**FIG. 1.**
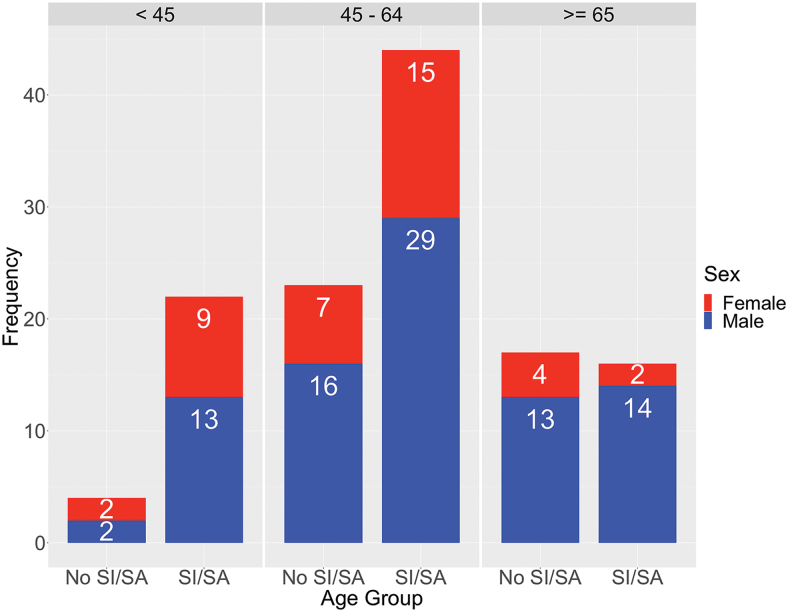
Age and sex distribution of RWC program participants across all 15 cohorts with 87 males (in *blue*) and 39 females (in *red*), separated by a lifetime history of suicide. The majority of at-risk veterans, 54% with a suicide history (ideation or attempt, SI/SA) are in the 45–64 age group, and secondarily 27% are in the under 45 age group. RWC, Resilience and Wellness Center; SA, suicide attempter; SI, suicide ideator. Color images are available online.

Participants' baseline characteristics and group differences are presented in Table 2. Data are presented for 15 cohorts, totaling 126 veterans who have completed the program, with 31% being female. Among the female participants, 65% had a positive screen for military sexual trauma (MST) in their EMR during service, as compared with 9% of male participants. Approximately 17.5% of participants have had a high-risk flag for suicide in their EMR at least once in the past 5 years. Across the cohorts, 65% (*N* = 82) had a lifetime history of suicide ideation or attempt (see suicide history distribution by age and sex; Fig. 1). Because prior suicide attempt is a prominent risk factor for future suicidal behavior,^112^ program engagement and treatment outcomes pre- and post-RWC program completion were investigated by using three groups, comprising veterans with a history of suicide attempt or ideation, and no history of ideation or attempt, as these groups potentially reflect the spectrum of risk severity.

**Table 2.** Demographics Data Representing 126 Participants in the Program

	Total	SA	SI	Nonsuicide (no SI/SA)	Group comparison,* p*-value
*N*	126	41 (33%)	41 (33%)	44 (35%)	
Age (years)	55.73 ± 12.45	52.54 ± 13.28	55.83 ± 12.80	58.61 ± 10.77	0.1890
Sex					
Male	87 (69%)	27 (66%)	29 (71%)	31 (70%)	0.8646
Female	39 (31%)	14 (34%)	12 (29%)	13 (30%)	
Service connection (yes)	87 (69%)	28 (68%)	34 (83%)	25 (57%)	0.0336
Depression (yes)	50 (40%)	17 (41%)	15 (37%)	18 (41%)	0.8841
PTSD (yes)	66 (52%)	24 (59%)	23 (56%)	19 (43%)	0.3100
Substance use (yes)	4 (3%)	2 (5%)	0 (0%)	2 (5%)	0.5455
SMI (yes)	21 (17%)	11 (27%)	8 (20%)	2 (5%)	0.0189

No significant group differences were observed by age, sex, and diagnosis of PTSD or depression. However, group differences was observed for diagnosis of serious mental illness (SMI, schizophrenia, psychosis or bipolar disorder) and service connection.

SA, suicide attempter; SI, suicide ideator; SMI, serious mental illness.

### Primary mental health outcomes

**FIG. 2.**
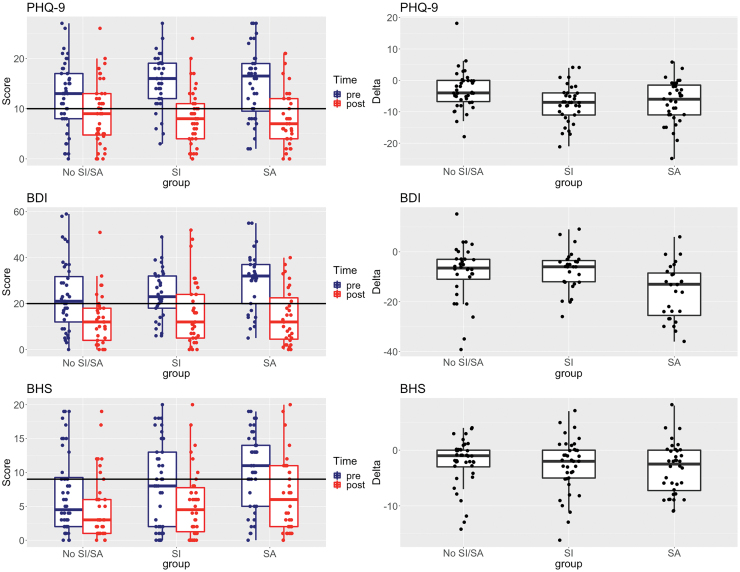
Mental health outcomes, including depressive symptoms (assessed by PHQ-9 and BDI) and hopelessness (assessed by BHS) shown by group, with ideators (SI), attempters (SA), and those with no suicide history (No SI/SA). *Left panels* show unwinsorized scores pre- versus post-RWC program completion, where clinically relevant moderate depressive and hopelessness symptoms are demarcated by *horizontal lines* at 10, 20, and 9 for PHQ-9,^82^ BDI,^83^ and BHS^84^, respectively. *Right* plots show intraindividual differences in scores [delta = (post − pre)] values. BDI, Beck Depression Inventory; BHS, Beck Hopelessness Scale; PHQ-9, Patient Health Questionnaire-9. Color images are available online.

Improvements for depression (via reduction in BDI/PHQ-9 scores) and hopelessness (via reduction in BHS scores) were observed by group relative to suicide history (Fig. 2 and Table 3). We also quantified the magnitude of the treatment effect by using Cohen's *d*, and we found that the RWC intervention resulted in medium to large treatment effects in reducing depressive and hopelessness symptoms (Table 4), with greater treatment gains within suicide groups. A significant reduction in suicidal ideation was observed at completion of the RWC intervention for both suicide groups (BDI/item-9: *p* = 0.0129 and PHQ-9/item-9: *p* = 0.0016; one-sided Wilcoxon signed-rank test).

**Table 3.** RWC Program Outcomes for Mental and General Health Assessments

	N	Total	SA	SI	Nonsuicide (no SI/SA)
		Mean* ± *SD or median [IQR] (adjust* p*-value)			
PHQ-9	109	−5.88 ± 6.11 (*p* < 0.0001)	−6.85 ± 6.81 (*p* < 0.0001)	−7.51 ± 5.84 (*p* < 0.0001)	−3.43 ± 4.94 (*p* = 0.0007)
BDI	86	−10.15 ± 9.79 (*p* < 0.0001)	−16.11 ± 11.07 (*p* < 0.0001)	−7.19 ± 7.65 (*p* = 0.0003)	−7.63 ± 8.12 (*p* = 0.0001)
BHS	114	−2.47 ± 4.11 (*p* < 0.0001)	−3.33 ± 4.61 (*p* = 0.0003)	−2.55 ± 4.46 (*p* = 0.0021)	−1.63 ± 3.07 (*p* = 0.0066)
PCL	68	−6.15 ± 14.44 (*p* = 0.0011)	−10.02 ± 12.72 (*p* = 0.0121)	−7.72 ± 17.01 (NS)	−2.93 ± 12.96 (NS)
BAI Scale	69	−4.65 ± 10.23 (*p* = 0.0005)	−7.11 ± 9.72 (*p* = 0.0129)	−6.61 ± 8.16 (*p* = 0.0021)	−1.67 ± 11.49 (NS)
PSS	68	−4.03 ± 7.87 (*p* = 0.0002)	−5.14 ± 10.35 (NS)	−5.46 ± 7.13 (*p* = 0.0021)	−2.37 ± 7.04 (NS)
MoCS Scale	66	6.23 ± 8.85 (*p* < 0.0001)	11.04 ± 7.35 (*p* = 0.0002)	7.18 ± 8.82 (*p* = 0.0021)	3.01 ± 8.51 (NS)
DVPRS^a^	70	−0.50 [1.44] (*p* = 0.0016)	−0.50 [1.38] (*p* = 0.0083)	−0.88 [1.56] (*p* = 0.0275)	−0.25 [2.19] (NS)
PSQI	105	−0.71 ± 1.87 (*p* = 0.0003)	−0.52 ± 1.80 (NS)	−0.94 ± 2.00 (*p* = 0.0126)	−0.64 ± 1.81 (NS)
ESS^a^	65	[6.00] (NS)	−1.50 [4.75] (NS)	0.50 [5.00] (NS)	[7.00] (NS)
HD	100	2.01 ± 6.95 (*p* = 0.0052)	1.93 ± 7.06 (NS)	1.88 ± 6.77 (NS)	2.19 ± 7.21 (NS)

Outcome measures with pre vs. post differences are shown as mean delta scores by group, with all *p*-values reported corrected for multiple testing via Benjamini-Hochberg method.

^a^ Non-parametric tests were used for DVPRS and ESS because of the skewed data distribution.

BAI, Beck Anxiety Inventory; BDI, Beck Depression Inventory; BHS, Beck Hopelessness Scale; DVPRS, Defense Veterans Pain Rating Scale; ESS, Epworth Sleepiness Scale; HD, Healthy Diet; IQR, interquartile range; MoCS, Measure of Current Status; NS, not significant; PCL, PTSD Checklist; PHQ-9, Patient Health Questionnaire-9; PSQI, Pittsburgh Sleep Quality Index; PSS, Perceived Stress Scale; SD, standard deviation.

**Table 4.** Magnitude of Treatment Outcomes and Effect Sizes (Cohen's *d*)

	Magnitude of treatment effect (Cohen's* d*)			
	Total	SA	SI	No SI/SA
PHQ-9	−0.96 (Large)	−1.01 (Large)	−1.28 (Large)	−0.69 (Medium)
BDI	−1.04 (Large)	−1.45 (Large)	−0.94 (Large)	−0.94 (Large)
BHS	−0.60 (Medium)	−0.72 (Medium)	−0.57 (Medium)	−0.53 (Medium)
PCL	−0.43 (Small)	−0.79 (Medium)	−0.45 (Small)	−0.23 (Small)
BAI	−0.45 (Small)	−0.73 (Medium)	−0.81 (Large)	−0.14 (Negligible)
PSS	−0.51 (Medium)	−0.50 (Medium)	−0.77 (Medium)	−0.33 (Small)
MoCS Scale	0.70 (Medium)	1.50 (Large)	0.81 (Large)	0.35 (Small)
PSQI	−0.38 (Small)	−0.29 (Small)	−0.47 (Small)	−0.36 (Small)
HD	0.29 (Small)	0.27 (Small)	0.28 (Small)	0.30 (Small)

Magnitude of treatment outcomes and effect sizes (Cohen's *d*) where values of *d*, correspond to negligible (−0.2<d<0.2), small (0.2 ≤ d < 0.5; −0.5 < d ≤ −0.2), medium (0.5 ≤ d < 0.8; −0.8 < d ≤ −0.5), and large effect size beyond. Only outcome measures for which the scores met normality assumptions are shown.

### Secondary mental health outcomes

Significant improvements in PTSD symptoms (via reduction in PCL scores in attempters) and generalized anxiety symptoms (via reduction in BAI scores in ideators and attempters) were found (Table 3 and Supplementary Fig. S1). For the measures of perceived stress and coping skills, we found a significant reduction in perceived stress only among the ideators (via decrease in PSS scores), and improved stress coping skills (via increase in MoCS Scale scores) in both suicide groups (Table 3 and Supplementary Fig. S2). Median to large RWC treatment effects on anxiety, PTSD symptoms, stress, and coping skills were found, again with greater gains among suicide groups (Table 4).

### General health primary and secondary outcomes

**FIG. 3.**
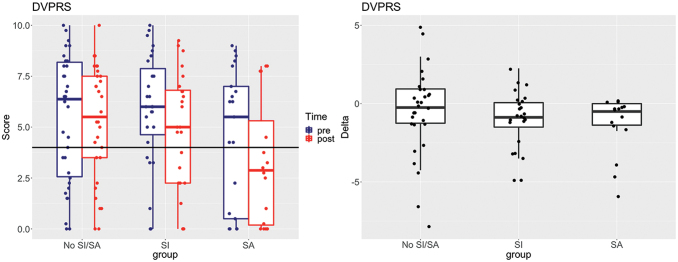
Pain outcomes assessed by the DVPRS shown by group, with ideators (SI; *n* = 24), attempters (SA; *n* = 16), and those with no suicide history (No SI/SA; *n* = 30). *Left panels* show unwinsorized scores pre- versus post-RWC program completion, where clinically relevant moderate pain symptoms are demarcated by *horizontal lines* at 4 for the DVPRS.^98^ *Right* plots show intraindividual differences in scores [delta = (post − pre)] values. DVPRS, Defense Veterans Pain Rating Scale. Color images are available online.

Significant improvements in pain were reported, specifically across the participants with a prior history of suicide ideation or attempt, who showed a reduction in pain symptoms via the DVPRS scores (Table 3 and Fig. 3). Overall, there were no appreciable improvements in measures of sleep quality, daytime sleepiness, or diet (Table 2 and Supplementary Fig. S3).

### Program engagement and sustainability

**FIG. 4.**
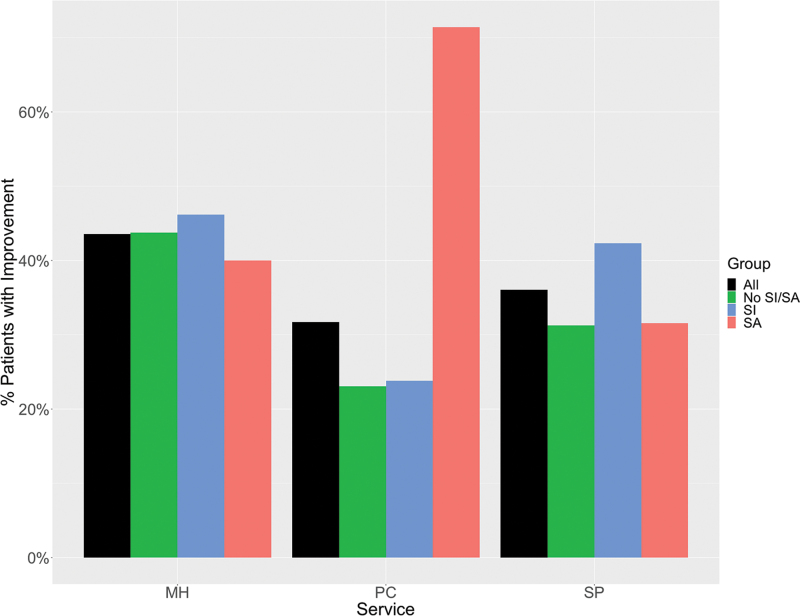
Percentage of patients who showed improvement in no-show rates across services comparing 3 months pre- and 3 months post-RWC program completion. (MH, mental health; PC, primary care; SP, specialty services, e.g., dermatology, rehabilitation medicine, cardiology, dental care, *etc.*). Color images are available online.

There were no differences in the retention rate among the three groups (Fisher's exact test; *p* = 0.7990). Attendance, quantified as the proportion of days attended out of the total sessions possible, showed significant group differences, yet all still above 84% in all three groups (nonsuicides = 95%, ideators = 95%, attempters = 84%; *p* = 0.0052); however, when examining weekly attendance over time, the group difference was no longer significant, showing a shared group-wise decline over time (*p* < 0.0001). Also, no disparity in group cohesion or connectedness by suicide history was found, which was measured at the last week of the RWC program assessed by the Group Cohesion Scale^43^ (Kruskal Wallis *p* = 0.8624). Moreover, to assess the potential sustainability of the RWC CIH intervention, using short-term preliminary data, we examined no-show rates (defined as the number of missed medical appointments) 3 months pre- and post-RWC program participation (based on data available for nine cohorts, *N* = 71). More than a 20% reduction in no-show rates was found across mental health, primary care, and specialty care services (Fig. 4), with suicide attempters showing the largest decline in no-show rates for primary care service visits.

### Program experience and evaluation

At the RWC program completion, participant feedback was obtained, with data showing that more than 90% of veterans reported feeling providers treated them with respect, that the program was easy to follow, and that the RWC program fulfilled the VA's commitment to them. On the Program Outcome Evaluation, greater than 75% of veterans endorsed “knowing how to eat better,” “feeling more connected to their fellow Veterans,” and “being more mindful,” and more than 96% would recommend the program to fellow veterans.

## Discussion

Whole systems multimodal CIH approaches^113–115^ have proven effective in complex clinical presentations in treatment of, for example, pain^116^ and PTSD.^117,118^ The RWC whole systems multimodal CIH intervention also shows significant effectiveness in reducing suicide ideation and its associated mental and general health symptoms. Specifically in this study, significant improvements were seen in mental health outcomes, including depression and hopelessness, PTSD and anxiety, stress and coping skills, and chronic pain from baseline to the 4-week RWC program conclusion, especially among the at-risk participants with a history of suicide ideation or attempt (Tables 3 and 4). In such multimodal CIH interventions as the RWC, it is difficult to tease apart what treatment contributed to improved outcomes, but an argument could be made that it is the whole systems approach itself that is crucial. Indeed, behavioral interventions that do not account for the multitude of risk factors and the complex phenotypic and lifestyle profiles of the at-risk individuals will likely not prove effective in reducing suicide risk. The RWC's multimodal CIH approach is a novel avenue, because to date no single treatment modality has been conclusively shown to adequately address the myriad of risk factors within the at-risk populations in suicide prevention.

A key strength of this treatment paradigm is that it is not based on the traditional clinician-driven diagnostic treatment and care. In line with the National Strategy to Prevent Veteran Suicide, the RWC is patient-driven, providing healthy living skills-based CIH interventions to at-risk participants to promote overall wellness. This was evident by the remarkable participant engagement and retention, with high attendance rates across all participants, even among those difficult to retain population of veterans with a history of suicide ideation (attendance rate of 95%) or attempt (attendance rate of 84%). Comparatively, the reported dropout rates of evidence-based psychotherapeutic approaches are 30%–60%.^119,120^ Also, preliminary data on a subset of participants show that these CIH interventions may have a sustained effect, based on reduction in no-show rates examined 3 months pre- and 3 months post-completion of the RWC program (Fig. 4). Notably, for those participants with a history of suicide ideation or attempt, there was an overall improvement of 71% no-show rate, in those who typically are a difficult-to-engage complex patient population with comorbid mental and medical conditions. Although the 3 months' time point potentially reflects short-term effects, the data are highly encouraging in demonstrating subject engagement and warrant future investigation to determine the long-term sustainability of an intensive, RWC CIH multimodal intervention.

This work has a number of limitations. First, since this was a program evaluation with no randomized control group, observed improvements may have been nonspecific to the RWC intervention, including potential placebo effects. Second, all outcome measures were based on validated self-report assessments, lacking objective measures including physiological parameters (e.g., blood pressure, heart rate, body mass index, and plasma cytokine/cortisol levels), as well as lacking direct measures to capture veteran social engagement. Third, although preliminary analysis showed decline in no-show rates 3 months post-RWC (Fig. 4), longitudinal assessments of mental and general health at 3/6 months follow-up may be warranted in future studies to establish long-term clinical utility. Fourth, RWC participation did not yield appreciable improvements in the veterans' sleep quality and diet, as interventions in these areas may have been too time-limited to be effective. Insomnia, the most prevalent behavioral sleep disorder among veterans,^46^ is represented in the majority of veterans in this study, who suffer from clinically significant sleep problems (Supplementary Fig. S3). Hence, future studies that deliver targeted interventions (e.g., CBT for insomnia^121^), in conjunction with RWC, might lead to clinically significant improved outcomes in sleep parameters. Overall, the demonstrated effectiveness of RWC presented in this program evaluation related to participant engagement and outcome improvements provides the necessary foundation on which to build future randomized clinical trials.

## Conclusions

Given patients' and veterans' preference to receive CIH therapies,^27,122,123^ especially among female veterans,^124^ the RWC provides a non-stigmatizing environment that appears to appeal to female veterans (31% female participants vs. 8% VA wide^125^), of whom the majority (65%) experienced MST. This potentially offers a novel avenue of care for the vulnerable female veterans, particularly those with MST exposure. As veterans, RWC participants receiving health care at the VA have a greater incidence of depression, PTSD, suicidal behavior, and life stressors (e.g., homelessness, unemployment, and disability) than the general population. Within this transdiagnostic context, the RWC intervention is associated with significant improvements in pain, depression/anxiety symptoms, and suicidal ideation for at-risk veterans who have a variety of health and life challenges, underscoring the significance of immersive, multimodal CIH approaches for suicide prevention.

## Supplementary Material

Supplemental data

Supplemental data

Supplemental data

Supplemental data
