# Supplementary material for: Effectiveness of Complementary and Integrative Approaches in Promoting Engagement and Overall Wellness Toward Suicide Prevention in Veterans
Source: J Altern Complement Med. 2021 Mar 31;27(Suppl 1):S-14–27. doi: 10.1089/acm.2020.0245 (PMC8035924; doi:10.1089/acm.2020.0245)
Supplement: Supplemental data [file Supp_Table1.docx]

|  | **Monday** | **Tuesday**  **(concurrent)** | **Tuesday**  **(concurrent)** | **Wednesday** | **Thursday** | **Friday** |
| --- | --- | --- | --- | --- | --- | --- |
| **9:30 –**  **10:00a** | Morning Mindfulness | Acupuncture  Individual sessions start at 8:30 | Morning Mindfulness | Morning Mindfulness | Morning Mindfulness | Morning Mindfulness |
| **10:00 –**  **11:00** | Exercise | Acupuncture | Sleep Hygiene | Exercise | Spirituality | Improvisation Class |
| **11:00 –**  **12:00p** | Nutrition | Acupuncture | Financial Literacy | Cooking Program | Pro Vetus Introduction | Exercise |
| **12:00 –**  **1:00p** | Lunch | Acupuncture | Lunch | Lunch | Lunch | Lunch |
| **1:00 –**  **2:00p** | Interpersonal Effectiveness | Acupuncture | Emotional Freedom Technique | Narrative Therapy | Next Steps | Yoga |
| **2:00 –**  **2:15p** | Steps for Tomorrow & Wrap Up | Acupuncture | Steps for Tomorrow & Wrap Up | Steps for Tomorrow & Wrap Up | Steps for Tomorrow & Wrap Up | Steps for Weekend  & Wrap Up |

Supplementary Table S1 - RWC 1 week Representative Class Schedule
