# Supplementary material for: Effectiveness of Complementary and Integrative Approaches in Promoting Engagement and Overall Wellness Toward Suicide Prevention in Veterans
Source: J Altern Complement Med. 2021 Mar 31;27(Suppl 1):S-14–27. doi: 10.1089/acm.2020.0245 (PMC8035924; doi:10.1089/acm.2020.0245)
Supplement: Supplemental data [file Supp_Fig1.docx]

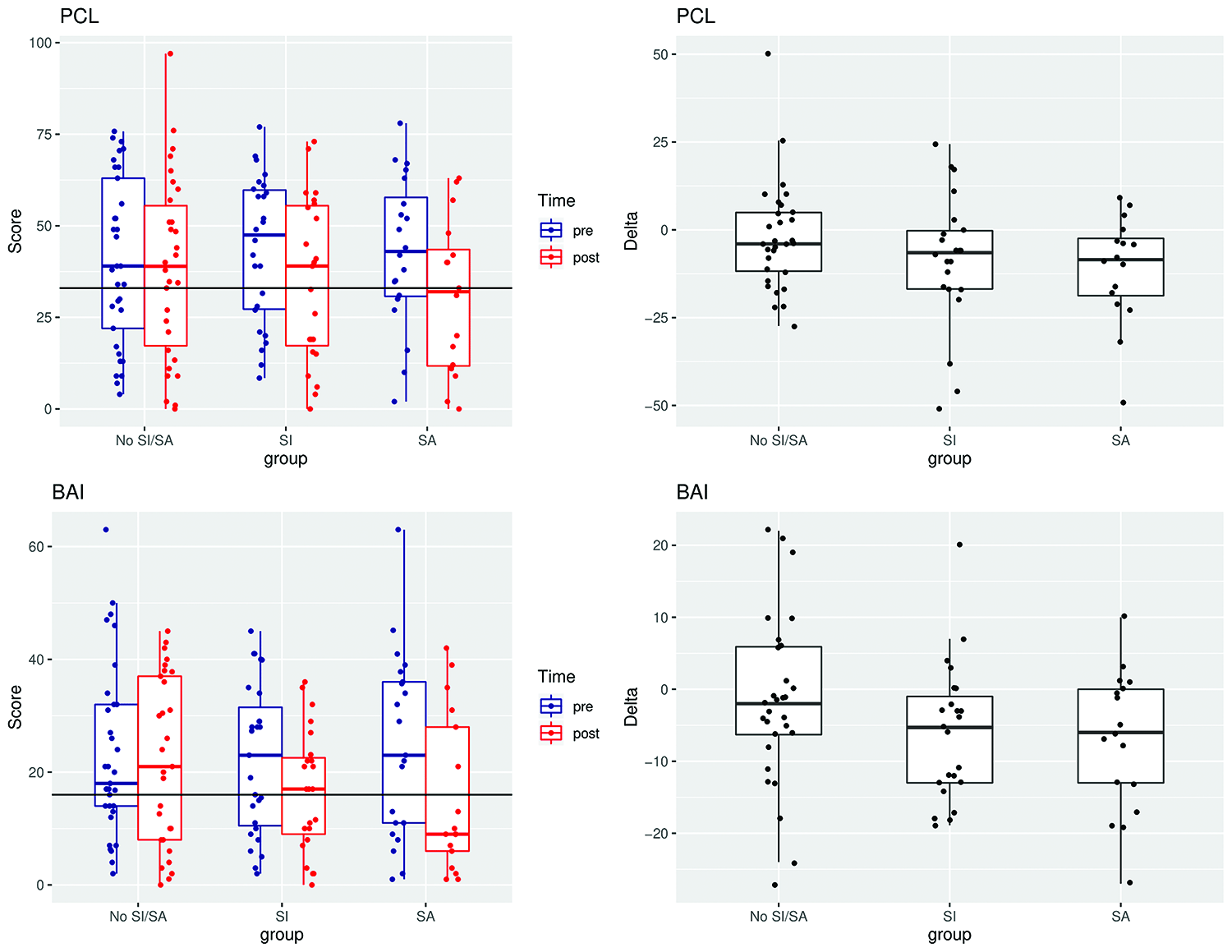


Figure S1: Anxiety outcomes assessed by PCL and BAI shown by group, with ideators (SI), attempters (SA), and those with no suicide history (No SI/SA). Left panels show unwinsorized scores pre vs. post RWC program completion, where clinically relevant moderate PTSD/anxiety symptoms are demarcated by horizontal lines at 33 and 16 for PCL^116^ and BAI^117^, respectively. Right plots show intra-individual differences in scores [delta=(post-pre)] values.
